# Efficient and flexible representation of higher-dimensional cognitive variables with grid cells

**DOI:** 10.1371/journal.pcbi.1007796

**Published:** 2020-04-28

**Authors:** Mirko Klukas, Marcus Lewis, Ila Fiete

**Affiliations:** 1 MIT Department of Brain and Cognitive Sciences, Cambridge, Massachusetts, United States of America; 2 Numenta, Redwood City, California, United States of America; University College London, UNITED KINGDOM

## Abstract

We shed light on the potential of entorhinal grid cells to efficiently encode variables of dimension greater than two, while remaining faithful to empirical data on their low-dimensional structure. Our model constructs representations of high-dimensional inputs through a combination of low-dimensional random projections and “classical” low-dimensional hexagonal grid cell responses. Without reconfiguration of the recurrent circuit, the same system can flexibly encode multiple variables of different dimensions while maximizing the coding range (per dimension) by automatically trading-off dimension with an exponentially large coding range. It achieves high efficiency and flexibility by combining two powerful concepts, modularity and mixed selectivity, in what we call “mixed modular coding”. In contrast to previously proposed schemes, the model does not require the formation of higher-dimensional grid responses, a cell-inefficient and rigid mechanism. The firing fields observed in flying bats or climbing rats can be generated by neurons that combine activity from multiple grid modules, each representing higher-dimensional spaces according to our model. The idea expands our understanding of grid cells, suggesting that they could implement a general circuit that generates on-demand coding and memory states for variables in high-dimensional vector spaces.

## Author summary

Entorhinal grid cells in mammals are defined by the periodic arrangement of their firing fields and play a major role in the representation of 2D spatial information. At the same time, they represent a variety of non-spatial cognitive variables. It is thus natural to ask what kinds and dimensions of variables it is theoretically possible for grid cells to represent. We show that grid cells can provide a neural vector space for unambiguous integration, memory, and representation of a variety of different (Euclidean) variables of much higher dimension than two, without requiring higher-dimensional grid-like responses. The same circuit can flexibly represent variables of different dimensions, doing so with high dynamic range and at low neural cost, without any reconfiguration of the recurrent circuitry.

## Introduction

It is widely believed that entorhinal grid cells in mammals play a central role in the representation of spatial information. But recent evidence indicates that grid cells are more versatile than initially assumed and also represent cognitive variables other than (self-)location in physical space. Grid cells respond to the location of visual gaze [1–3], the locus of covert attention [4], or the values of two parametrically varied features of cartoon bird images [5]. In all these cases, the recorded cells exhibit a response structure that matches that of grid cells during spatial exploration, with single unit recordings in rodents indicating that the same grid cells are reused across variable types [6, 7]. This suggests that all of these variable types are represented by a single population of grid cells, which underlie very general types of cognitive representation. All these examples involve 2-dimensional (2D) variables. However, cognitive variables are not limited to two dimensions, and it is a natural question to consider what kinds and dimensions of variables, theoretically, it is possible for grid cells to represent.

At the same time grid cell responses are structurally and dynamically constrained. Across a range of novel, familiar, and distorted spatial environments [8], during navigation on sloped terrains [9, 10] or one-dimensional tracks [11], and most strikingly, across sleep states when the animal receives no external spatial inputs and is rather driven by presumably high-dimensional internal spontaneous activity [12, 13], grid cells are confined to a fixed set of states with preserved cell-cell correlations that match those measured during awake exploration in familiar 2D spatial environments. The fact that grid cells conserve the pairwise firing relationships they exhibited in their spatial responses directly suggests that the dynamics of a grid module are confined to a 2D set of states that is invariant across time, task, and behavioral state. Even the physical layout of grid cells in the brain is organized in a grid-like topographical pattern [14, 15] that mirrors—and likely drives—the functional response of grid modules. But how generally useful can the grid code be, if the autonomous states of each grid module are inherently 2-dimensional?

We propose a coding scheme for high-dimensional variables that is consistent with these structural and dynamical constraints and assume that the activity of each grid module remains confined to a 2D toroidal attractor in the associated neural state space.

From a purely mathematical viewpoint, the possibility of encoding higher-dimensional variables using grid cells is not surprising—after all the combined state space of multiple (*M*) 2-dimensional toroidal attractor manifolds, formed from the 2D grid responses of the *M* individual modules, is already a high-dimensional (2*M*-dimensional) toroidal manifold.

However, the current literature does not offer any concrete coding schemes that exploit this fact. On the contrary, existing proposals and experimental searches center around the formation of individual grid modules that individually support high-dimensional grid responses [16, 17]. These models face two major problems. The first is a question of resources. The formation of high-dimensional modules is costly, running head-on into the curse of dimensionality: The number of cells needed to form a single stable *N*-dimensional continuous attractor network with *K* resolvable states per dimension is ∼ *K*^*N*^ while the same state capacity can be achieved by ∼ *NK* cells by forming *NK* lower dimensional attractor networks representing one dimension each.

The second is a question of flexibility. The recurrent connectivity of an attractor network must be tailored to the dimension and geometry of the attractor manifold and cannot be easily reconfigured on demand. The construction of a 3*D* grid requires entirely different connectivity from a 2*D* grid. This is particularly problematic if the actual dimension of the input might vary; what if the circuit encodes a variable that appears to be low-dimensional but eventually turns out to vary in more dimensions than initially expected, or if the circuit must represent variables of different dimensions at different times? For the same circuit to alternate between representing a 2D and 3D variable would require a full rewiring of the recurrent circuit at each alternation, for which there is no known mechanism that is reasonably fast.

In addition to solving the problems of efficiency and flexibility in representing variables of different dimensions, we will show that our proposed coding scheme exhibits a smooth and automatic handoff in the allocation of coding states toward additional dimensions based on demand and toward increasing the coding range per dimension when the number of dimensions shrinks, all without changing previously assigned codewords or recurrent connectivity. These properties are enabled by combining the power of nonlinear mixed selectivity with compositional modular representations in grid cells.

## Results

Modular codes, in which a set of neurons is divided into a number of disjoint groups, each dedicated to encode different aspects of the represented variable, enable a high-dimensional state space for a cheap number of cells; thus avoiding the “curse of dimensionality” we mentioned in the introduction. This can obviously be exploited for the representation of higher-dimensional variables but relies on a pre-partitioning depending on variable dimension.

Perhaps less widely known, the immense capacity of modular codes can alternatively be leveraged for the representation of a fixed, low-dimensional variable, and produce a massive library of unique coding states. The high-level idea is that the joint coding space can be efficiently packed with a well-folded lower-dimensional manifold to produce a very large coding range. The prime example of the use of this strategy in the brain is the modular grid cell system. The grid code is efficient on two levels, capacity (it utilizes a sizable fraction of the available coding space) and fast mapping of input to representation (as opposed to a slow learned lookup table). It is flexible to a limited extent: we know that the same circuit is used for 1d and 2d variables.

The ability to encode either dimensions or range per dimension raises the interesting question of whether modularity can be exploited simultaneously for both, and whether a coding scheme exists that can flexibly hand-off excess capacity in range for dimension and vice versa, without reconfiguration (or pre-partitioning). We show below that the answer can be affirmative, by combining properties of mixed representations with modular codes in grid cells.

To quantify the capacity of our model, we define the *coding range* of a code to be the maximal side-length of a hypercube of dimension *N* over which no two points are assigned *similar* grid codes. We consider two codes to be similar if their distance falls below a previously fixed threshold Δ (see Methods for details), which represents a finite coding resolution, consistent with encoding and reading out a variable using a population of neurons with noisy responses; it can be thought of as the inverse square-root of the Fisher information about phase in neural spike counts across the grid modules [18].

The remainder of the section is organized as follows: We start with a brief review of grid cells. From there we proceed with the definition of two distinct coding schemes, illustrating efficiency and flexibility of a code, followed by the presentation of our numerical results. We conclude the section with a characterization of our model’s tuning curves.

### The grid code in 2D

**Fig 1:**
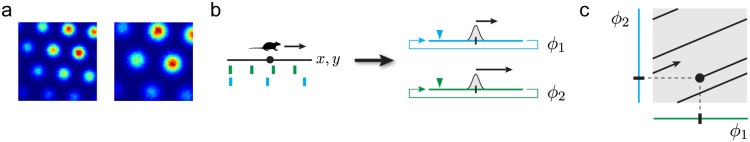
**(a)** Firing fields and their hexagonal arrangement shown for two simulated grid cells from two modules of different scale. **(b)** Schematic picture of the origin of periodic firing fields. Left: a schematic environment (black line) with cell responses (green, blue) of two grid cells from two modules of different scale. Right: schematic picture of two grid modules depicted as 1-dimensional circular continuous attractors. The colored triangles symbolize recording devices whose responses are shown on the left. A positional change (small arrow on left-hand side) corresponds to a change in phase (small arrows on right-hand side) in each module. Both phase-changes are related by a fixed scalar factor resulting in different spatial periodicity. **(c)** Schematic picture of the coding space (gray box) spanned by multiple, here 2, grid modules (blue and green). The modulo-arithmetic nature of the grid code enables an extraordinarily huge coding range by tightly “packing/folding” an environment (black line) into code space (gray). The fixed ratio of module scales results in a linear embedding with fixed “slope”.

Mammalian grid cells are defined by their periodic firing fields in planar environments: they fire at multiple locations corresponding to the vertices of an equilateral triangular lattice (Fig 1a). A grid module is a discrete sub-network of grid cells with common underlying lattices (same period and orientation) that differ only by translational shifts. The firing fields of all the cells within a module uniformly cover the entire space such that at any time some sub-population within the module is active. A grid module represents the animal’s time-varying location *x*(*t*) as a 2D phase *ϕ*(*x*) [18]. Combining the phase information from multiple modules produces an unambiguous positional code over an extremely large range [18–20] that is exponentially bigger than the scale of the individual lattice periods, with the exponent growing in proportion to the number of modules. We refer to the combined activity of multiple distinct modules, or equivalently the set of phases, as a grid code; Fig 1 provides a schematic picture illustrating the key mechanism and properties of the “classic” grid code.

The internal state in a module *ϕ* is updated as [21–24]:

$$\begin{array}{c} \frac{d\phi }{dt}=A\cdot \frac{dx}{dt}. \end{array}$$ (1)

Here $A\in R^{2\times 2}$ is a linear operator that governs how the animal’s velocity $\frac{dx}{dt}$ in 2D space is mapped to changes in the internal 2D phase. The phases are obtained by forming the quotient of the Euclidean plane (the co-domain of *A*) and a hexagonal lattice Λ. Mechanistically, these phase updates can be implemented in a recurrent continuous attractor network with feedforward velocity inputs. Once the module is anchored, by assignment of a particular phase *φ*_0_ to a specific position *x*_0_ in the environment (e.g. through place cells or other spatially-specific cells [25, 26]), the circuit will automatically generate a grid code (phase) for any other location *x* in the environment reached via a path *γ* connecting *x* and *x*_0_. If *x* lies in Euclidean space, the assigned phase is guaranteed to be independent of the particular path, ensuring a well-defined code for *x* regardless of trajectory:

$$\begin{array}{c} \phi \left(x\right)=\varphi_{0}+\int A\cdot \frac{d\gamma }{dt}\;dt=\varphi_{0}+A\left(x-x_{0}\right)\;\;\;\left(\text{mod}\;\Lambda \right). \end{array}$$ (2)

The responses of different modules during 2D spatial navigation can be generated by a simple scalar gain variation that premultiplies a common operator *A*, according to models as well as (still accruing) empirical support [8, 24, 27].

The grid cell coding description above applies immediately to the local representation of arbitrary (locally Euclidean) 2D variables *x*, not just 2D spatial position. The only required change to represent a new cognitive space is the construction of a separate feedforward projection *A* mapping velocities in the external space to the velocity inputs of the grid modules. Under this view, existing grid cell models that integrate velocity inputs [24, 28] can already explain how the same network can represent both spatial and non-spatial 2D variables [1–7] without reconfiguration of the recurrent circuit, merely by changing the velocity operator *A* that feeds into the grid cell modules. This idea will be key for our model to form high-dimensional representations. But first let us take a look at another conceptually simpler model to set a baseline for the performance of our model and to highlight key properties of our model.

### Disjoint modular grid code: An efficient baseline for our model

We can now return to the question of cell-efficient encoding of variables of higher dimensions with 2D grid cells. We first present a conceptually simple disjoint modular grid coding scheme that is efficient for both dimensionality and range, yet inflexible.

For simplicity, assume that the number of modules, *M*, is a multiple of *N*, the dimension of the encoded variable *x*. Divide the modules into *N* disjoint groups of *M*/*N* modules, and let each group separately encode a single coordinate of *x*, so the problem has been decomposed into the task of separately encoding *N* variables of dimension 1 each (manifold factorization). The coding range *L* will then be determined by the minimum coding range of one of the groups, that is

$$\begin{array}{c} L=\text{min}\{L_{1},\ldots ,L_{N}\}, \end{array}$$ (3)

where *L*_*i*_ (*i* = 1, …, *N*) denotes the 1-dimensional coding range of the *i*th group.

We know from previous theoretical work that if the population response in each grid module in some time-bin determines position as a spatial phase with resolution Δ (meaning that the intrinsic uncertainty in estimating phase from the population response is Δ), and if all grid periods are distinct but have a similar spatial scale (magnitude), which we denote as λ, then the 1-dimensional coding range per group scales as (cf. [18] and S1B Fig):

$$\begin{array}{c} E\left(L_{i}\right)\;\propto \;\lambda \cdot \Delta \cdot (\frac{1}{\Delta^{2}}{)}^{M/N}. \end{array}$$ (4)

The expectation value is taken over random choices of how to “slice” each 2D grid module to represent one component of *x* [11]. When *M* is a multiple of *N*, we can compute the empirical distribution for the *L*_*i*_ (S1a Fig) as well as an expected value for the overall coding range *L*, which we will come back to later. We can also obtain a coding range when *M*/*N* ≥ 1 is not an integer by interpolating between the distributions of *L*. The disjoint modular grid code thus enables representation of an *N* > 2-dimensional variable with a coding range per dimension that increases exponentially with the number of modules. Thus, unlike a code based on the construction of high-dimensional grids, the above scheme is efficient with regards to cell-number *and* coding range.

However, it must be constructed for the specific dimension of the input (this determines how the modules are grouped, and once the modules are grouped, the range per dimension is fixed). If the dimensionality of the encoded variable shrinks, modules must be reallocated to reflect this change before the encoding range per dimension can increase. Hence, the code is not *flexible*, in contrast to what we propose next.

### Mixed modular grid code: Our model

In the previous section we exploited the modular structure of the grid code in two consecutive steps. We first formed different groups each dedicated to encode a single coordinate of the input variable (multiple periodic modules assigned to each coordinate ensure an efficient disambiguation of position along that dimension over a large range), and then leveraged the groups as disjoint modules encoding different 1-d inputs (to efficiently represent higher-dimensional variables), resulting in a code that is efficient but inflexible. We are now going to merge these two steps such that *each* grid module receives a linear combination of all input velocity components, and not just one coordinate—this can be viewed as a form of mixed selectivity [29] generalized to a set of analog rather than discrete variables. Though the mixing of input velocity components is linear, the periodic code itself is nonlinear, resulting in nonlinear mixed selectivity which we will show is important for flexibility.

**Fig 2:**
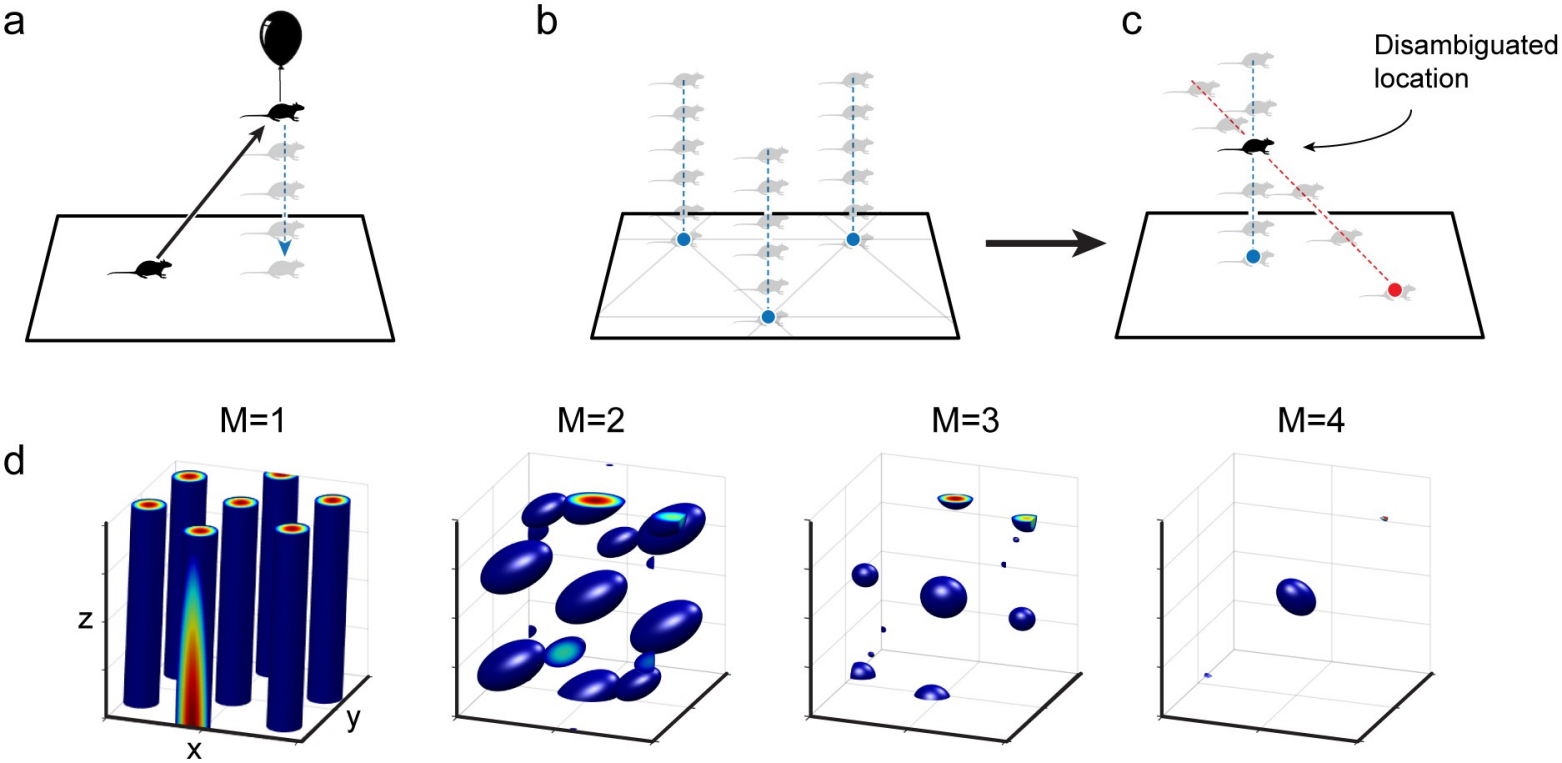
**(a)** If the encoded variable is 3D (here, the animal leaves the 2D plane), simple projection down to the 2D phase is ambiguous and consistent with multiple locations in the z-direction. **(b)** There are two sources of ambiguity, the periodicity of the grid code within the *xy*-plane, and the ambiguity in the z-direction. **(c)** Two different 2D phases for two modules are set by two distinct projections (red and blue) of the 3D value onto a plane. Together they are able to simultaneously resolve both sources of ambiguity. **(d)** Estimates of the value of the encoded 3D variable obtained by combining the ambiguous estimates of 1, 2, 3, and 4 modules as in (c). Given the cell responses we compute a probability estimate and show only areas that exceed a fixed threshold (blue blobs). The spacing between the blobs defines the coding range, that is, the range over which the code is unique. With an increasing number of modules the range quickly grows larger than the individual periods.

The central observation of this paper is that just as multiple modules operating independently to integrate velocity solve the problem of the ambiguity of representation by periodic responses, they can *simultaneously* solve the ambiguity that results from the compression of higher-dimensional inputs to two-dimensional responses: Suppose that the grid cell system constructs *M* distinct and independent projection operators *A*_*α*_ (of size 2 × *N* each). Each operator projects *N*-dimensional velocities from an *N*-dimensional space into a 2D signal for input to one of the *M* grid modules. These velocity signals are not related simply by a scalar gain, as for 2D spatial responses, but must differ more fundamentally in their responses to each input dimension (Fig 2c). We choose *A*_*α*_ to be independent random projections for each module. These *M* independent projections can be viewed as a single matrix of size 2*M* × *N*, which is of full-rank almost surely if *N* ≤ 2*M*. This implies that the intersection of the kernels of all projections is trivial, consisting solely of the zero vector. In consequence they compress different portions of the input space and can mutually resolve their ambiguities (Fig 2c and 2d). With realistic estimates for the number of grid modules (*M* = 4, …, 8) the code could represent variables of dimension as large as *N* = 8, …, 16, Fig 2c and 2d. We will now turn our attention to the coding range of our model and show that mixed random projections allow us to leverage the modular structure of the code for both dimension and range.

### Efficiency of our model

Recall that we described the coding range of the disjoint modular grid code for *N*D variables in terms of the coding range for a 1D variable. Though shown to be exponential in how the 1-dimensional coding range scales with number of modules, it cannot be analytically computed because it depends on number-theoretic interactions between the different real-valued periods and finite phase resolution [18, 19]. Similarly, we cannot obtain an analytical expression for the coding range over which a mixed multi-module code can provide unambiguous (unique) code-words.

However, we can numerically compute how the code scales by using the following approach [18]: Begin at some arbitrary point *x*_0_ in the *N*-dimensional external variable space, and assign this point to a fixed phase *ϕ*_0_ in the joint coding space of all modules (because the grid code is translation-invariant, this choice does not incur a loss of generality). Next, center a box around *x*_0_, and expand this box progressively along all *N* dimensions, checking for when any of the points at the frontiers of the box first maps back into a Δ-ball of the starting phase *ϕ*_0_ (Methods). When such a “collision” first occurs, the code has reached its capacity, and no longer supplies unique code-words for the encoded variable. The side-length of the box, just before the collision, corresponds to the coding range as described earlier.

**Fig 3:**
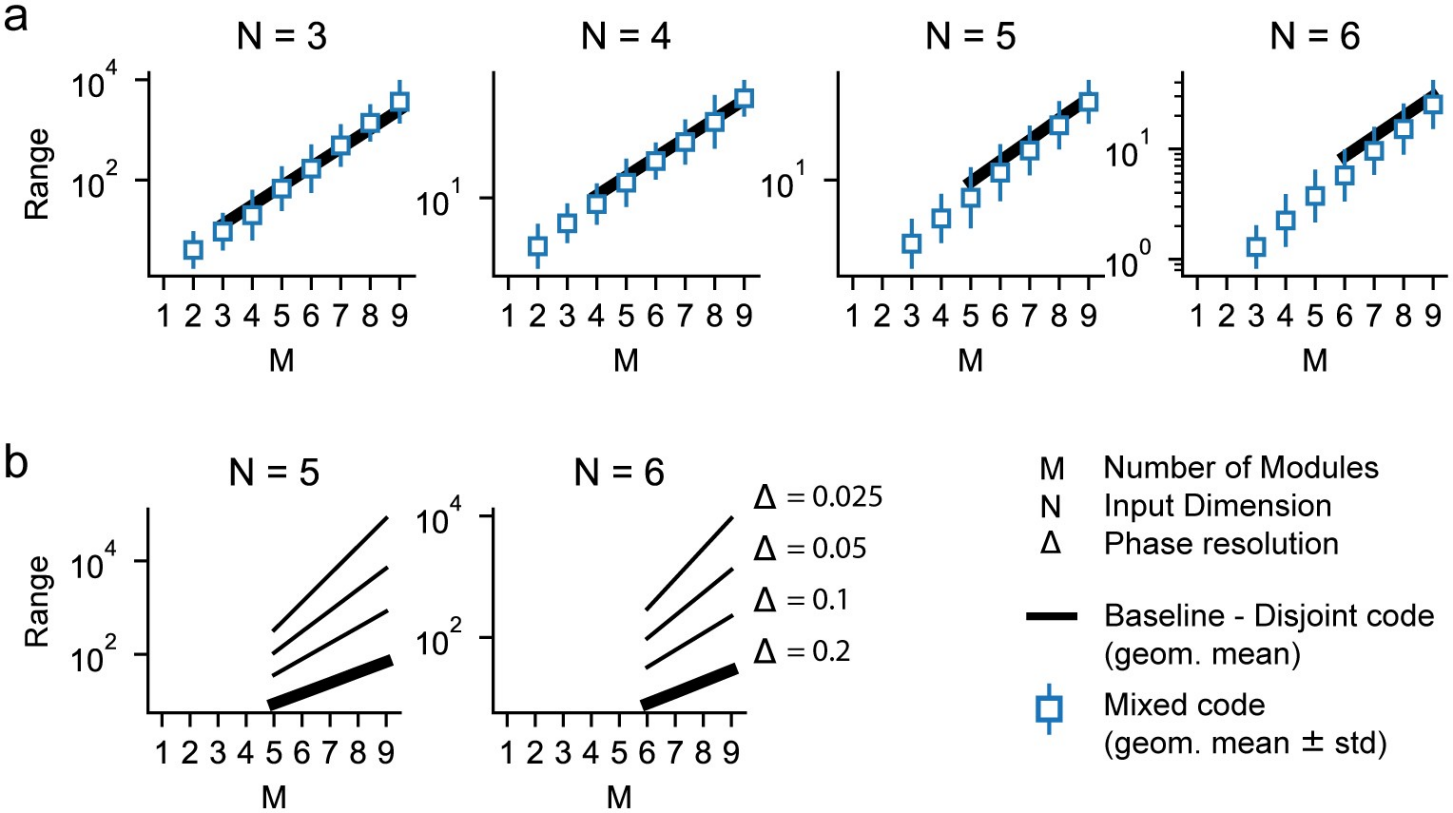
Capacity grows exponentially with module number. All plots show the *dynamic* coding range of our model (see Methods section). **(a)** Exact coding range of the grid code for variables of dimension 3 to 6, assuming an overly conservative phase resolution of Δ = 0.2 to reduce time of computation. We show the geometric mean and standard deviation over 1000 different draws of the projection matrices *A* for each pair *M*, *N*. The entries of the matrices are sampled independently from a standard normal distribution. To compute the expected value E(W) of the benchmark in Eq 3, we also run this simulation with *N* = 1 (S1 Fig), solid line. The capacity grows exponentially with the number of modules; the benchmark provides an estimate of the expected capacity. **(b)** We use the benchmark to show the coding range for more realistic values of phase resolution (Δ = 0.2, …, 0.025). We chose the benchmark rather than measuring the exact range for practical reasons (the run-time scales with the *volume* of the coding range not its side-length). Results shown for *M*/*N* ≥ 1.

In this way, we numerically compute the coding range as a function of the number of encoding grid modules (*M* = 1, …, 9) for input variables of various dimensions (*N* = 3, …, 6), Fig 3a (white squares show the mean value over different random samples of the projection matrices). Because of our emphasis on flexibility, the projections operators *A*_*α*_ are always of dimension 2 × 6 and the projection matrices are held fixed even as the input dimension *N* is varied between 1 and 6 (though only the corresponding sub-matrix of dimension 2 × *N* is used when *N* < 6). We find that the mixed modular grid code generates unique code-words for variables in high dimensions, and does so over ranges per dimension that far exceed the individual periods, Fig 3a. As we will show through a conceptual argument and plot later, linear mixing together with nonlinear periodic responses are important for the functionality of the code. In fact, the coding range per dimension clearly grows exponentially with the number of modules, regardless of variable dimension. More specifically, the exponential growth of coding range per dimension is in the *excess dimension* of the representation, which we define as 2*M* − *N*. This rate of exponential growth in the mixed modular scheme closely matches that of the disjoint modular scheme, Fig 3a, illustrating its efficiency.

For reasons of computational complexity, our primary numerical calculations are performed with a rather conservative (low) phase resolution (Δ = 0.2). To gain a more realistic picture of model performance with finer phase resolution, we consider the dependence of coding range on phase resolution for a moderate number of modules and dimensions (S2 Fig) and also consider how the disjoint modular grid code range, which serves as an efficiency benchmark for the mixed modular grid code, changes with phase resolution (Fig 3b). Consistent with [18], range grows as a power of phase resolution, regardless of the dimensionality of the encoded variable. Plausible phase resolutions, combined with exponential scaling of range in the excess dimension, lead to very large ranges per encoded dimension. Next, we consider the model’s flexibility.

### Flexibile tradeoff between range and dimension

**Fig 4:**
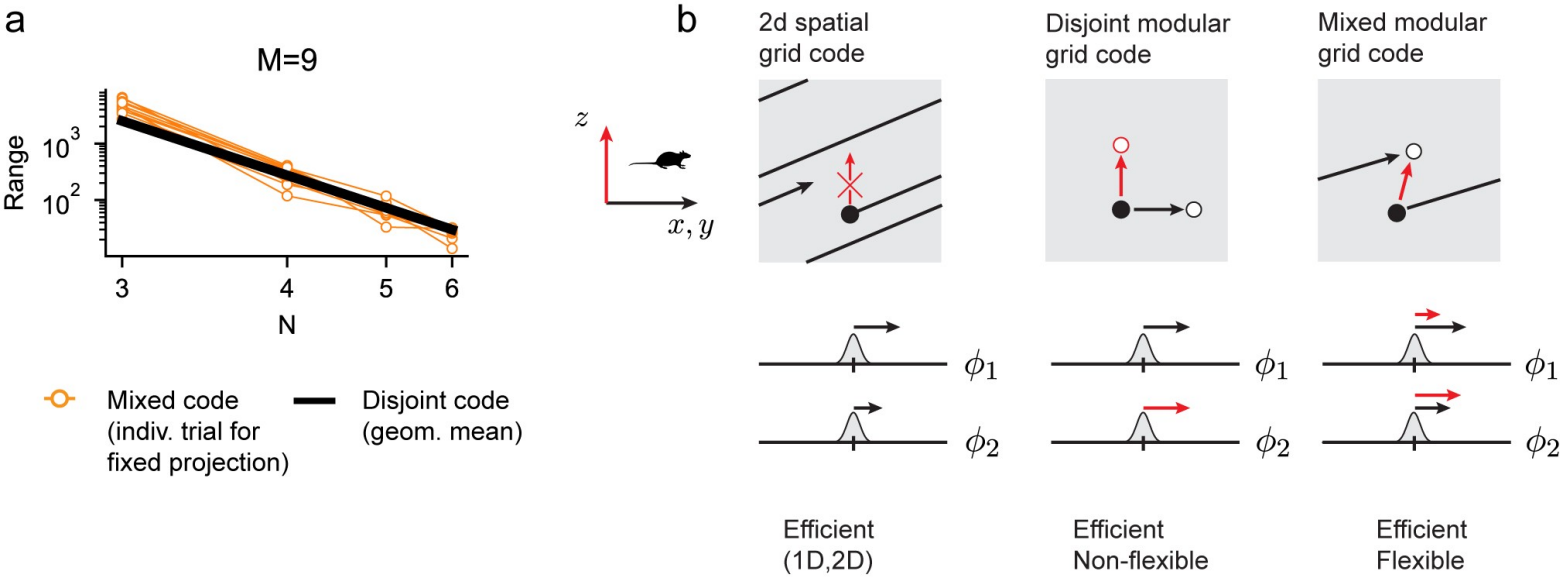
Flexibility of the mixed modular grid code: Conceptual explanation. **(a)** Change of coding range while increasing the dimensionality of the input (*N*) and keeping the projection fixed (per trial), illustrating the flexibility of the scheme (10 trials are shown). **(b)** **Left**: The conventional grid code encodes only 2D variables (*x*, *y*, black arrows; and not *z*, red); movements in *x*, *y* result in phase changes across modules, but changes in *z* do not. These phase changes evolve along only one direction in the coding space (here, showing 2 modular phases), governed by the ratios of the grid periods. **Middle**: The disjoint grid module code: Modules are partitioned into disjoint groups, one set coding *x*, *y*, the other coding *z*. Changes in *x*, *y* update one set of phases, changes in *z* another; the red circle cannot be reached from the black by only changing *x*, *y*, without changing *z*. **Right**: Updates in module activity are decoupled as for the disjoint code but *each* module participates in the representation of *all* input dimensions. The periodicity of the code makes it possible to reach (and thus use) the white coding state from the black in two ways: by moving along *x*, *y*, or along *z* (red and black arrows).

When we fix the random projections *A*_*α*_, but decrease the dimensionality of the input variable, the same projection appropriates states previously allocated to encoding different dimensions to encoding a larger range per dimension, as can be seen because the coding range grows as the input dimension is decreased, Fig 4a. The conceptual reason for the flexibility of the mixed modular grid coding scheme is illustrated in Fig 4b (right panel).

First, consider the conventional 2D grid code for 2D variables (x,y): starting at some point in the multi-modular coding space (Fig 4b, black dot in left panel), it is only possible to move along a specific direction to move through the coding space—different modules are constrained to change phases by a fixed proportion, given by the ratios of their periods. Thus, there is only one way to reach an unused coding state (Fig 4b, white dot in left panel), which is to keep moving an increasing distance along the 2D input space until that coding point is reached.

In contrast, consider the disjoint modular grid code. Here, some modules are entirely given over to representing a different dimension (*z*) in the input space. Starting from some point in coding space (Fig 4b, black dot in middle panel), the only way to reach another point that is offset along the *z*-devoted module (Fig 4b, red dot in middle panel) is to move along *z* in the input space. It is not possible to reach and thus use this point in coding space by increasing displacements in *x*, *y*. States cannot be traded to exchange coding range for coding dimension, thus such codes are not flexible.

Finally, consider the mixed modular code. As in the disjoint modular grid code, updates in different modules are decoupled, but this time *each* module participates in the representation of *all* input dimensions. The mixed projections together with the periodicity of the code, an essential nonlinearity, makes it possible to connect (thus use) two coding states in different ways, slicing through both represented range and dimension: by moving along a different input dimension or continuing along the same dimension (Fig 4b, right panel, red and black arrows, respectively). In this way, modules are neither exclusively allocated to specific input coordinates or dimensions, nor exclusively allotted to produce a given range for each dimension. The full coding space can be used by changes in either property (dimension or range per dimension) of the input variable, and the same space can be used interchangeably by both.

### Predicted tuning curves for *N*-dimensional representations

Will it be possible to identify whether grid cells can and do perform flexible representation of high-dimensional variables? For the grid cell system to work according to our model, different modules have to be capable of changing their internal states independently of each other, through the action of separate velocity projection operators. A tantalizing hint that this is possible appears in [30], where different grid modules in the same individual animal appear to rescale by different amounts in response to an environmental deformation.

A key signature of our proposed scheme involves differences in tuning curves across modules. Even after recording neural responses in higher-dimensional spaces, it remains practically difficult to characterize tuning curves in higher dimensions. However, characterizing the high-dimensional responses by plotting the tuning curves along any 2D subspace of the explored *N*-dimensional input space already provides highly diagnostic information. The basic prediction of a mixed modular grid code is that when encoding higher-dimensional variables, different modules will exhibit differently shaped tuning curves that do not look simply like scaled versions of each other. Specifically, the different modules will be lifts of some 2D grid along some plane, but the planes along which each module looks like a regular grid will differ from each other. Thus, along a common 2D subspace, the tuning of cells in different modules will look like differently oriented random slices of a high-dimensional lift of a 2D grid.

**Fig 5:**
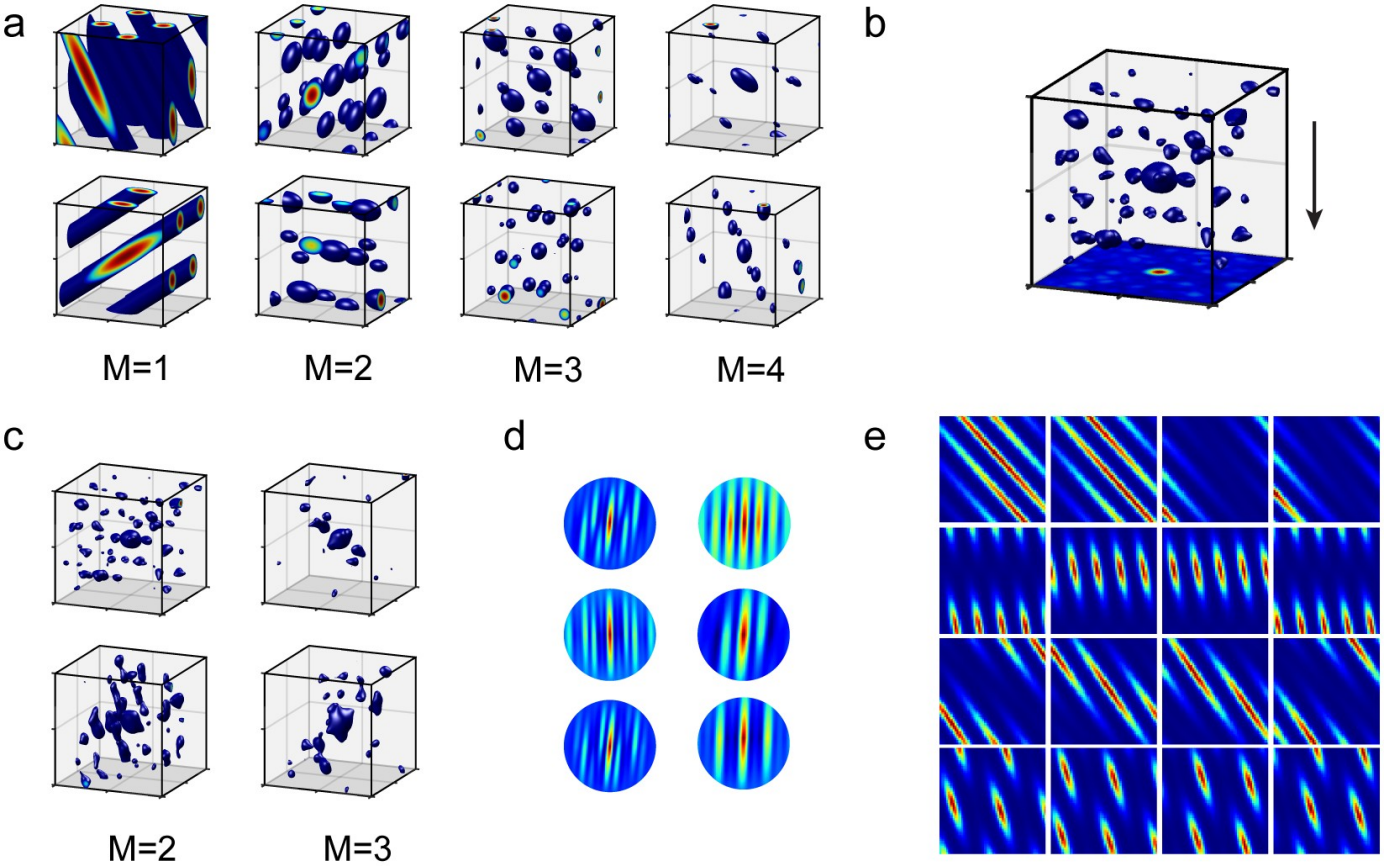
Predictions about grid cell firing. For ease of illustration, we consider here the encoding of a variable in three dimensions. **(a)** Left-most column (*M* = 1): 3D tuning curves of two grid-cells from different modules using our coding model. Remaining columns (*M* > 1): 3D tuning curves of two conjunctive cells reading from *M* different modules using our coding model. **(b)** 3D tuning curve of a conjunctive cell. The xy-plane shows a projected tuning curve taking the maximum along the z-axis. **(c)** Realistic tuning curves implemented by simulating multiple continuous attractor grid cell network modules with noisy neural activity. **(d)** Auto-correlations of grid responses along a vertical plane according to our model. The auto-correlations resemble those recorded from rats climbing a “pegboard” in [9]. **(e)** Each row shows the 2D responses of 4 co-modular cells over a randomly chosen tilted plane (shown on the left in gray) in 3D space. Different rows correspond to different modules and the modules encode space according to our model.

For instance, if the input variable is 3D, the 3D tuning curves in different modules are different lifts of a 2D grid. A 3D lifted response of a 2D grid consists simply of elongated fields along one direction, consistent with empirical findings in [9]. The responses of cells in different modules along a common 2D subspace will look like differently oriented slices through this lifted response (Fig 5a, *M* = 1), which will then resemble distorted grids (Fig 5e), ranging from perfectly equilateral triangular grids to non-grid-like and relatively complex, for instance, bands of bands (Fig 5e, 2nd row). (In the atypical case where the 2D subspace exactly aligns with one of the null or lift directions of a module, those cells will have periodically arranged stripe responses; see S3 Fig for a broader sampling of possible grid cell response geometries).

Within a module, the responses of different cells are generated from translations of the tuning of the module (each row of Fig 5e shows co-modular responses over 2D slices). If plotted over a large enough area, these translational relationships will be apparent, but when plotted over smaller areas, they need not appear as simple shifts of a canonical 2D response pattern (e.g. Fig 5e, top row), similar to the relationships seen in co-modular cells in 1D environments which are generated by cutting a lower-dimensional slice through translations of a higher-dimensional (2D) lattice [11]. Nevertheless, the common origins of the response of co-modular cells means that they are predicted to obey systematic invariant cell-cell correlation relationships across environments and variables of different dimensions.

Many cells in entorhinal cortex and hippocampus in bats and in some rat experiments express spatial fields in 3D environments that are less structured than grid cells. According to our model, if some entorhinal and hippocampal cells were combining inputs from two or three grid modules, these conjunction-forming cells would exhibit localized 3D fields with some regularity in spacing, but without full grid-like periodicity and thus no clear notion of a spatial phase (Fig 5a).

In sum, a central prediction of the mixed modular coding hypothesis in which the grid cell system could collectively and flexibly use its multiple modules to encode variables of higher dimension than two is that the projections to different modules should be different, and therefore that in such situations, the responses of grid cells in different modules will differ in the geometry of their tuning curves.

## Discussion

### Implications for computation

The multi-module representation of grid cells provides a pre-fabricated, ready-to-use, general high-dimensional neural affine vector space that can be used for both representation and memory of arbitrary vectors (of dimension ≤ 2*M*), and more specifically, for integration of vector inputs. (The term *affine* makes explicit the lack of a preferred “zero” element. Each point in the space admits a neighbourhood that naturally carries the structure of a vector space with the point at its origin.) The representation is efficient: it generates exponentially many states with linearly many neurons, thus solving the curse of dimensionality problem faced by more naive coding schemes (e.g. by the formation of unary codes or grids in higher dimensions). The update mechanism of grid cells permits vector-algebraic operations between the stored vectors, required for vector integration in higher-dimension abstract spaces. So long as displacements in the abstract spaces are provided as inputs to the network, the network can thus efficiently represent, hold in memory, and perform algebraic sum operations on general, abstract vectors of different dimensions without any reconfiguration of the recurrent grid cell network. We believe these results and implications fulfill, at least in theory, intuitive expectations that the very peculiar grid code might be extraordinary in the computations it enables.

### Observed 3D responses in grid cells, entorhinal cortex, and hippocampus

In some studies of animals exploring higher-dimensional spaces, specifically 3D spatial environments, the response of grid cells is elongated and undifferentiated along one dimension, while remaining grid-like in the other two [9]. This kind of tuning is consistent with our prediction, and we have shown it allows for unique coding along the third dimension if the projections (and thus the undifferentiated direction) are not aligned across modules.

Most of the field elongations recorded in [9] were close to perpendicular to the level ground, while mixed modular coding would predict elongations along different directions in different grid modules. It is unclear whether the specific setup of the experiment favored a strong prior bias favoring an internal 2D world interpretation, level with the physical ground, and whether in non-spatial cognitive navigation the same bias would persist. It will be important to systematically study across-module tuning in high-dimensional coding scenarios.

Recently, grid cell responses have been examined in bats flying through 3D environments. Bats crawling on 2D surfaces exhibit the same 2D triangular grid cell tuning [31] as rats and mice. In 3D, consistent with our theory, the responses seem not to clearly exhibit regular 3D grid patterns [32]. However, the fields do seem to be localized in all 3 dimensions, at least in the vicinity of a tree around where the bats forage for food—to our knowledge the cells have been recorded either exclusively during flight *or* during crawling. It is possible in this case that localized higher-dimensional fields are formed in the hippocampus or the lateral entorhinal cortex based on spatial landmarks. Alternatively, localized fields seen in medial entorhinal cortex and hippocampus in 3D could be formed by conjunctions of grid cells encoding higher-dimensional spaces according to our model, as shown in Fig 5a, which qualitatively matches some of the reported properties of entorhinal cells in flying bats. A similar situation might hold for the observed localization of fields in 3D, in rats navigating 3D wire mesh cubes [16]. However, an absence of band-like structure in grid cells along all dimensions during 3D coding would not be consistent with our theory.

### Mixed modular grid codes and mixed selectivity

The mixed modular grid code combines two powerful concepts: the compositionality of modular codes for high capacity and cell-efficiency, and mixed selectivity coding for flexibility in trading off the available coding capacity for use in either representing large ranges per dimension of the encoded variable or representing higher-dimensional variables, without any reconfiguration of the recurrent circuitry. It generalizes the concept of nonlinear mixed selectivity [29] to show how it can be used effectively for representing analog, metric Euclidean variables, with a special form of nonlinearity (periodic phases) that does not damage the ability of the code to represent, in a translation-invariant way, variables that are translation-invariant.

## Methods

### Numerical computation of capacity

Numerical computation of capacity for mixed modular grid code: Given the number of modules *M*, a maximum input dimension *N*_max_, and a phase resolution Δ, a single trial consists of sampling a random matrix *A*_*α*_ of size 2 × *N*_max_ for each module and computing the *coding range* *L* = *L*(*M*, *N*, Δ) for all input dimensions *N* ≤ *N*_max_ (in order to support the model’s flexibility). The entries of the matrices were independently sampled from a standard normal distribution.

Recall that a grid code consists of an ordered set of 2*d*-phases on a twisted torus. The distance of two grid codes is determined by the maximum of their component-wise distances. We determine the coding range of the code by computing the side length of a maximal collision-free cube centered at the origin of the encoded variable; see S4 Fig. Here we consider two grid codes to collide if their distance in coding space is smaller than or equal to half the phase resolution—this is based on the assumption that we only observe noisy samples from a distribution which is centered at the true phase and whose support is determined by Δ (S4 Fig; pink region on the right). However, in a small neighbourhood of the origin (moving along each dimension by an amount smaller than all the grid periods) the encoding map is one-to-one. Any point thus admits a small neighbourhood of points whose associated phases are closer than Δ (S4 Fig; pink region on the left); it is necessary to ignore these points while performing our search for collisions for the capacity computations. We therefore compute the minimal hyper-rectangle enclosing these ignored points and then incrementally extend this box outward to find the maximal collision-free rectangular regions. We measure the coding range in units determined by the size of this minimal hypercube; this is also referred to as *dynamic range* in the literature. In other words, we use the side-lengths of the minimal box as the units of measurement and adapt our coordinate system in the input space accordingly. In this adapted coordinate system both the minimal box and the maximal collision-free region are in fact cubes (S4 Fig).

The search for collision follows a divide-and-conquer approach that extends the search region and then subdivides the new frontier region into smaller pieces for which collisions with the origin can be computed deterministically. This means that our collision search is not based on sampling the high-dimensional input space and ensures that we do not miss *any* collision within the search region.

### Tuning curves

Idealized tuning curves were computed as follows: As an idealized attractor manifold we chose a twisted torus obtained by the quotient of the Euclidean plane and a hexagonal lattice Λ with basis $\left\{{\left(1,0\right)}^{T},{\left(\text{cos}\;\frac{\pi }{3},\text{sin}\;\frac{\pi }{3}\right)}^{T}\right\}$. For each module we randomly sampled a (2 × 3)-matrix *A*; we compute a module’s phase associated to *x* as *ϕ*_*x*_ = *A* ⋅ *x* (mod Λ). The rate map of an idealized grid cell at position *ϕ*_0_ (for convenience we chose *ϕ*_0_ = 0) within a module was then computed as

$$\begin{array}{c} g\left(x;A\right)≔\text{exp}(-d{\left(\phi_{x},\phi_{0}\right)}^{2}), \end{array}$$

where *d*(*ϕ*_*x*_, *ϕ*_0_): = min_λ∈Λ_ ‖*x* − λ‖_2_. The rate map of a conjunctive cell combining the activity of *m* idealized grid cells in distinct modules with projections *A*_1_, …, *A*_*m*_ was computed as

$$\begin{array}{c} c\left(x;A_{1},\ldots ,A_{m}\right)≔\sum_{i=1}^{m}g\left(x;A_{i}\right) \end{array}$$

The firing fields in Fig 5a, 5b and 5c were obtained by thresholding the rate map *c* of a conjunctive cell at *θ* = min *c* + 0.8 ⋅ (max *c* − min *c*) over a cube with side-length 2.

Realistic tuning curves were implemented by simulating multiple grid cell modules with noisy neural activity, as in [24], and driving these networks with randomly projected velocity inputs. The center of the encoded space was treated as a landmark that served to reset the grid phases to a correct value for that location, to avoid the accumulation of excessive errors. These more realistic tuning curves are shown in Fig 5b and 5c.

## Supporting information

S1 Fig 1-dimensional capacity. **(a)** Histograms of the 1D capacity data. Note that for each *M* the distribution is roughly log-normally distributed (1000 data-points for each *M*). For all computations the phase resolution is Δ = 0.2. **(b)** The 1D capacity of our randomized approach (blue error-bars). We show geometric mean and standard deviation of the data (1000 data-points for each *M*). The capacity grows proportional to the benchmark Δ ⋅ (1/Δ^2^)^*M*^ (thick black line); cf. [18].
(TIF)

S2 Fig Capacity as a function of phase resolution. Capacity grows as a power of phase resolution, regardless of the dimensionality of the encoded variable.
(TIF)

S3 Fig A zoo of firing fields. **(a)** A tilted plane (blue) in 3D space and 16 different projections (magenta lines) onto a common 2D input subspace (gray) along which the responses are an equilateral triangular lattice. **(b)** 6 × 6 different Firing fields on the blue plane induced by a family of 6 × 6 projections whose angles vary as indicated in (a).
(TIF)

S4 Fig Coding range. Left: Schematic picture of input space. Pink region contains points whose phase is in the Δ-neighbourhood of the associated phase on the right (pink box on the right). Right: Schematic picture of joint coding space of multiple grid modules. Black thick line represents the image of the box on the left hand side under the grid coding map. Black dots represent two encoded positions: the phase representing the origin in input space is surrounded by a Δ-neighbourhood (pink) of noise. The other dot illustrates a collision.
(TIF)
