# Supplementary figures and images for: Efficient and flexible representation of higher-dimensional cognitive variables with grid cells
Source: PLoS Comput Biol. 2020 Apr 28;16(4):e1007796. doi: 10.1371/journal.pcbi.1007796 (PMC7209352; doi:10.1371/journal.pcbi.1007796)

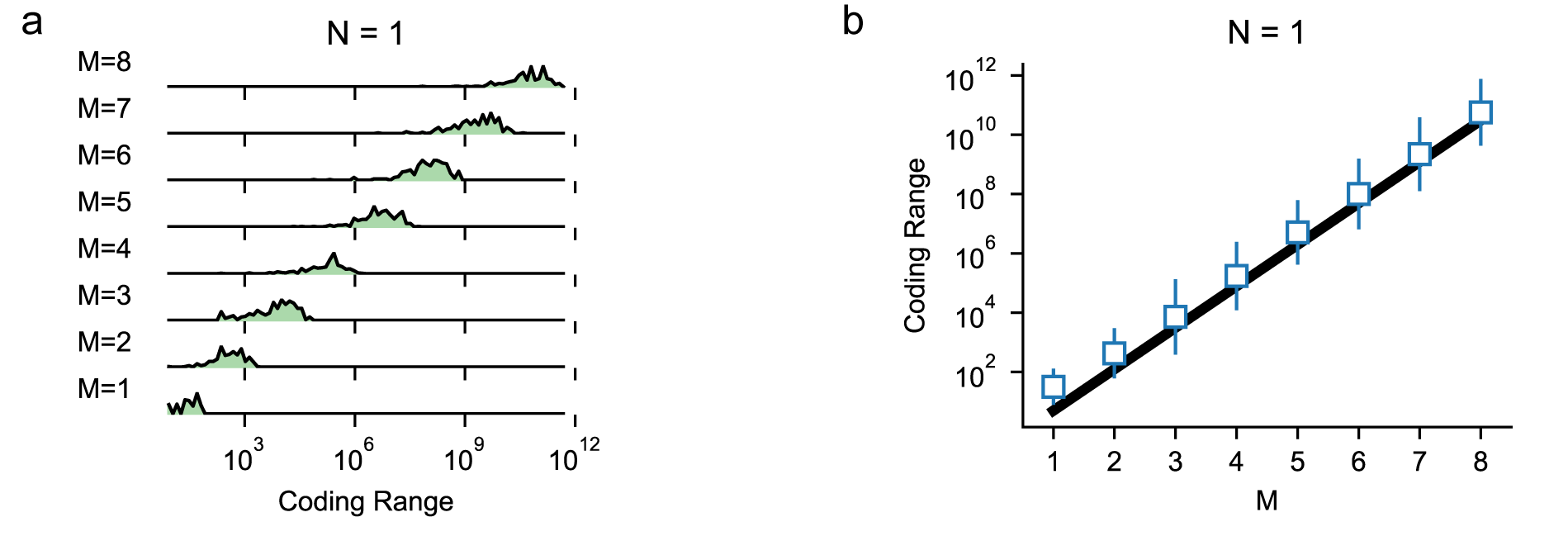

Supplement: S1 Fig — (a) Histograms of the 1D capacity data. Note that for each M the distribution is roughly log-normally distributed (1000 data-points for each M). For all computations the phase resolution is Δ = 0.2. (b) The 1D capacity of our randomized approach (blue error-bars). We show geometric mean and standard deviation of the data (1000 data-points for each M). The capacity grows proportional to the benchmark Δ ⋅ (1/Δ2)M (thick black line); cf. [18]. (TIF) [file pcbi.1007796.s001.tif]

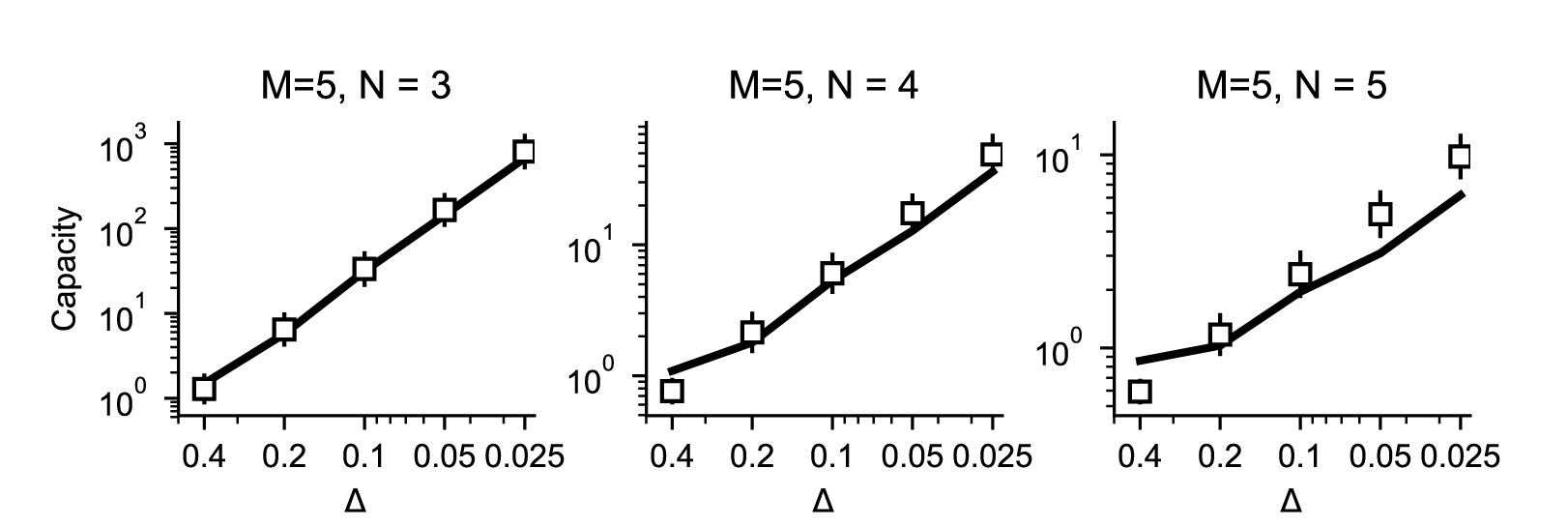

Supplement: S2 Fig — Capacity grows as a power of phase resolution, regardless of the dimensionality of the encoded variable. (TIF) [file pcbi.1007796.s002.tif]

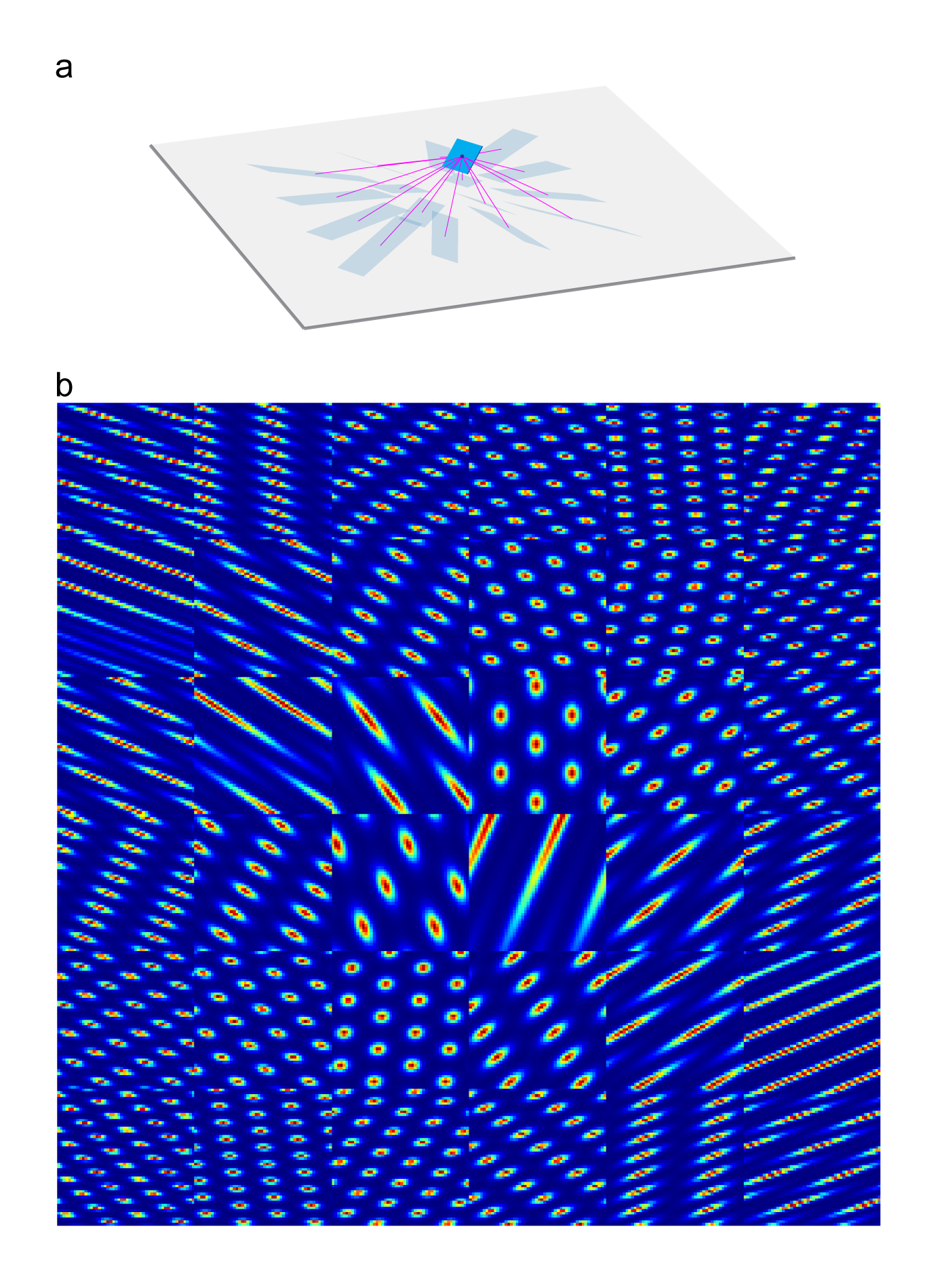

Supplement: S3 Fig — (a) A tilted plane (blue) in 3D space and 16 different projections (magenta lines) onto a common 2D input subspace (gray) along which the responses are an equilateral triangular lattice. (b) 6 × 6 different Firing fields on the blue plane induced by a family of 6 × 6 projections whose angles vary as indicated in (a). (TIF) [file pcbi.1007796.s003.tif]

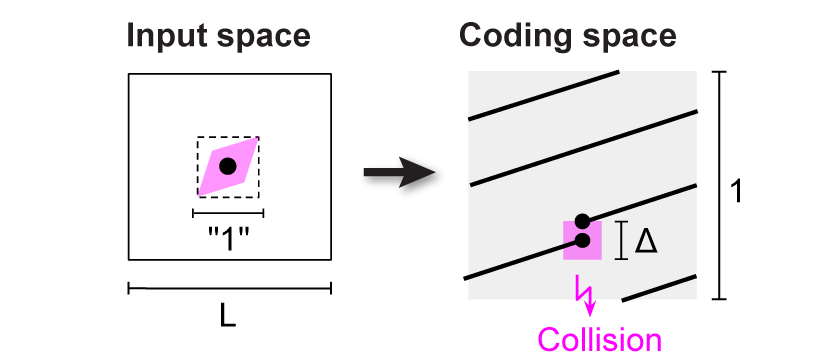

Supplement: S4 Fig — Left: Schematic picture of input space. Pink region contains points whose phase is in the Δ-neighbourhood of the associated phase on the right (pink box on the right). Right: Schematic picture of joint coding space of multiple grid modules. Black thick line represents the image of the box on the left hand side under the grid coding map. Black dots represent two encoded positions: the phase representing the origin in input space is surrounded by a Δ-neighbourhood (pink) of noise. The other dot illustrates a collision. (TIF) [file pcbi.1007796.s004.tif]
